# Pituitary Stalk Morphology as a Predictor of New-Onset Adrenocortical Insufficiency and Arginine Vasopressin Deficiency after Transsphenoidal Resections of Pituitary Macroadenomas: A Retrospective Single-Center Study with a Focus on iMRI

**DOI:** 10.3390/cancers15153929

**Published:** 2023-08-02

**Authors:** Ralf Becker, Michal Hlavac, Gwendolin Etzrodt-Walter, Fabian Sommer, Christian Rainer Wirtz, Bernd Schmitz, Andrej Pala

**Affiliations:** 1 Department of Neuroradiology, University of Ulm, Lindenallee 2, 89312 Günzburg, Germany; bernd.schmitz@uniklinik-ulm.de; 2 Department of Neurosurgery, University of Ulm, Lindenallee 2, 89312 Günzburg, Germany; 3 Endocrinological Center Ulm, Weinberg 41, 89075 Ulm, Germany; 4 Department of Otorhinolaryngology, University of Ulm, Frauensteige 12, 89075 Ulm, Germany

**Keywords:** pituitary adenoma, transsphenoidal surgery, intraoperative MRI, adrenocortical insufficiency, arginine vasopressin deficiency

## Abstract

**Simple Summary:**

Common complications of transsphenoidal surgery for pituitary macroadenomas include new-onset adrenocortical insufficiency and arginine vasopressin deficiency. New-onset adrenocortical insufficiency is especially challenging to detect early and entails prolonged therapy. We performed a retrospective, monocentric analysis of pituitary stalk morphology in intraoperative MRI during 48 transsphenoidal surgeries for macroadenoma. An intraoperative thickening of the pituitary stalk was identified as an easy-to-acquire predictor of these endocrinological insufficiencies and, thus, might aid early detection. Still, these findings need to be validated in future prospective studies.

**Abstract:**

Background: A new-onset adrenocortical insufficiency (NAI) is the most critical postoperative endocrinological complication after transsphenoidal surgery for macroadenomas. Because of increased mortality risk, arginine vasopressin deficiency (AVP-D) is also a relevant postoperative complication. This study aimed to identify easy-to-acquire magnet resonance imaging (MRI) aspects of the pituitary stalk to predict these insufficiencies after transsphenoidal surgery. Methods: Pituitary stalk morphology was reviewed intraoperatively and three months postoperatively in the MRIs of 48 transsphenoidal surgeries for macroadenomas. NAI was validated in endocrinological follow-up controls 10–14 months post-surgery. Results: Intraoperative pituitary stalk diameters were 0.5 mm larger in patients who developed NAI and AVP-D. The odds ratio was 29 for NAI and 6 for AVP-D in binary regression analysis. A value of 2.9 mm was identified as the optimal cut-off for the minimal pituitary stalk diameter regarding NAI, with a high specificity of 89%. There was no difference in pituitary stalk diameter regarding these insufficiencies three months post-surgery. Conclusions: We identified an increased pituitary stalk diameter in intraoperative MRIs as a predictive factor of NAI and AVP-D after transsphenoidal surgery. These findings might improve the early detection of NAI and, thus, optimal management. However, validating these retrospective findings in prospective studies is obligatory.

## 1. Introduction

Pituitary adenomas (PAs) arise in the anterior pituitary gland (PG) and account for up to 15% of all intracranial tumors and up to 90% of all sellar and parasellar space-occupying lesions [1]. Transsphenoidal surgery (TSS), first described in 1907 by Schlofer et al., is currently the primary surgical approach for PAs [2]. Because intraoperative magnet resonance imaging (iMRI) results in an increased rate of gross total resections in transsphenoidal adenomectomy, iMRI has been more frequently established in TSS in recent years [3]. Endocrinologically, because of the possibility of adrenal crisis, postoperative new-onset adrenocortical insufficiency (NAI) is the most relevant endocrinological complication in TSS [2]. Since 2–12% of all patients undergoing TSS and up to 18% of patients after microscopic transsphenoidal pituitary surgery develop NAI post-surgery, monitoring and treating this potentially life-threatening insufficiency is essential [2,4,5]. However, because common adverse events in glucocorticoid replacement therapy include osteopenia, weight gain, and cardiovascular disease, the superiority of either empiric substitution after TSS or substitution only in those patients developing evident NAI has not yet been shown [5,6,7]. Still, considering the risk of adrenal crisis, some patients are over-supplemented, and there is an ongoing effort to rapidly diagnose NAI via early-morning cortisol level measurements after TSS instead of delaying diagnostics until dynamic testing of the hypothalamic-pituitary-adrenal axis can be conducted using the insulin tolerance test (ITT), corticotropin-releasing hormone (CRH), or the Synacthen^®^ test 4–6 weeks post-surgery [2,8,9].

With a postoperative incidence rate of up to 45% for transient and up to 10% for permanent arginine vasopressin deficiency (AVP-D), this salt and water balance disorder is also a relevant complication after TSS given its association with possible complications of hypernatremia, like epileptic seizures, and increased mortality [10,11,12]. Furthermore, monetary aspects play a role in AVP-D because of the increased unplanned readmission frequency after TSS and prolonged inpatient stays [10,13,14]. Thus, because of the clinical relevance of NAI and AVP-D, this study aimed to identify easy-to-acquire (intraoperative) MRI parameters that can predict these insufficiencies after TSS for macroadenomas.

## 2. Materials and Methods

### 2.1. Patients

We performed a retrospective single-center study of 243 transsphenoidal surgeries for intrasellar tumors at the Department of Neurosurgery of the University Hospital Ulm at the BKH Günzburg site between January 2014 and December 2019. Only adult cases with histopathological evidence of PA, and intraoperative MRI and radiological evidence of macroadenoma were included in the final sample [15]. Surgical revisions due to complications such as CSF leakage or hemorrhages, partial resections, and surgery for recurring PA were excluded from the analysis. Further, cases undergoing resection of residual tumors after performing iMRI were excluded to ensure no further severe manipulation of the sella region with possible injury to the pituitary stalk, which, therefore, could not have been captured with iMRI. The approval of the ethics committee of the University Hospital Ulm was obtained (524/21), and the data were treated according to the Declaration of Helsinki standards.

### 2.2. MRI Data

**Figure 1:**
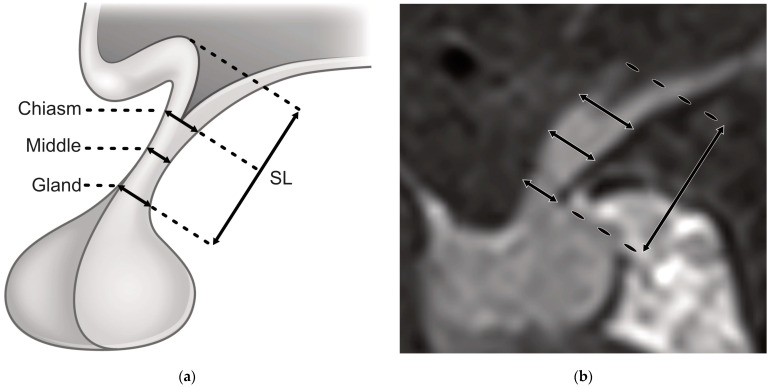
Illustration of MRI variables. (**a**) Illustration of the three different levels on which the sagittal pituitary stalk diameter was obtained and illustration of the length of the sagittal stalk (SL) as defined in this study; (**b**) Illustration of these variables in sagittal T1 contrast-enhanced intraoperative MRI.

Regarding radiological data, intra- and postoperative MRIs three months post-surgery had to be available for inclusion in this study. For iMRI, a 1.5T scanner (Espree, Siemens AG, Erlangen, Germany) was used at our neurosurgical department as a one-room solution (Brainsuite). Thin slice (2 mm) high-resolution coronal and sagittal T1 and T2 turbo spin echo (TSE) and post-contrast T1 (T1c) images were acquired. Corresponding sequences were used in postoperative MRI. However, postoperative MRI scanners were variable. DeepUnity Diagnost 1.2.0.1 (DH Healthcare GmbH, Bonn, Germany) with Xero Viewer (Agfa HealthCare N.V., Mortsel, Belgium) was used for image analysis. The MRI variables are listed in Table 1, including morphological definitions and imaging planes. The length of the PS was defined as the combined length of the infundibular recess and the sub-infundibular length of the stalk (Figure 1) to decrease inaccuracy due to spatial resolution. However, all pituitary stalk diameter measurements only refer to the sub-infundibular stalk. Acquisition of the MRI parameters was performed by a single radiologist from the neuroradiological department.

### 2.3. Endocrinological Data

All cases had complete endocrinological data on the endocrinological functioning of the PG available before surgery. NAI was defined as the absence of an increase in cortisol above 18.0 µg/dL in an ITT or CRH test 10–14 months post-surgery [16]. Hydrocortisone substitution was established with an administration of 100 mg intraoperatively, 80 mg on the first day, 70 mg on the second day, 40 mg on the third day, and 25 mg on all following days post-surgery until a functioning adrenocortical axis was evidenced in an ITT or CRH test 4–6 weeks or 10–14 months after TSS. (Transient) AVP-D was defined as a urine volume greater than 300 mL/h, increased serum sodium above 145 mmol/L, and a specific urine weight less than 1.005 [17].

### 2.4. Surgical Procedure

The head was placed slightly reclined in a 0° straight-ahead position in the MRI coil. During the study period, the endoscopic and microscopic transsphenoidal approach was performed in our department. The method was chosen independently of the patient based on the surgeon’s preference, with an increasing shift toward the endoscopic technique. Rigid 0°-, 30°-, and 45°-Hopkins endoscopes with the four-hand technique were used during the resection. The microscopic resection was performed using a direct unilateral trans-nasal para-septal approach. With the intraoperative impression of complete tumor resection, MRI imaging was performed. There was no subsequent re-resection after obtaining iMRI in the sample studied here since there was MRI evidence of total resection. All procedures were performed by senior physicians specialized in (endoscopic) transsphenoidal surgery.

### 2.5. Statistical Analysis

Statistical analysis was performed using SPSS 29 (Lead Technologies, Charlotte, NC, USA). Continuous data are provided as mean +/− standard deviation. For the comparison of continuous data regarding the prevalence of NAI and AVP-D, independent *t*-tests were used in the presence of statistical normal distribution. Normal distribution was evaluated using Kolmogorov–Smirnov, histogram, and Q-Q-plot. In addition, a *t*-test for paired samples was performed for a follow-up comparison of continuous data. Relevant variables identified with these tests were analyzed for statistically significant predictors with a logistic binary regression model using the inclusion method. Optimal cut-off values for the predictive factors were further analyzed using the Youden index on receiver operating characteristic (ROC) curves. A *p*-value of ≤0.05 was considered statistically significant. GraphPad Prism 9.51 (GraphPad Software, Boston, MA, USA) was used for box-plot diagrams. Artistic illustrations were produced with Inkscape 1.2 (Free Software Foundation, Boston, MA, USA).

## 3. Results

### 3.1. General Characteristics

**Figure 2:**
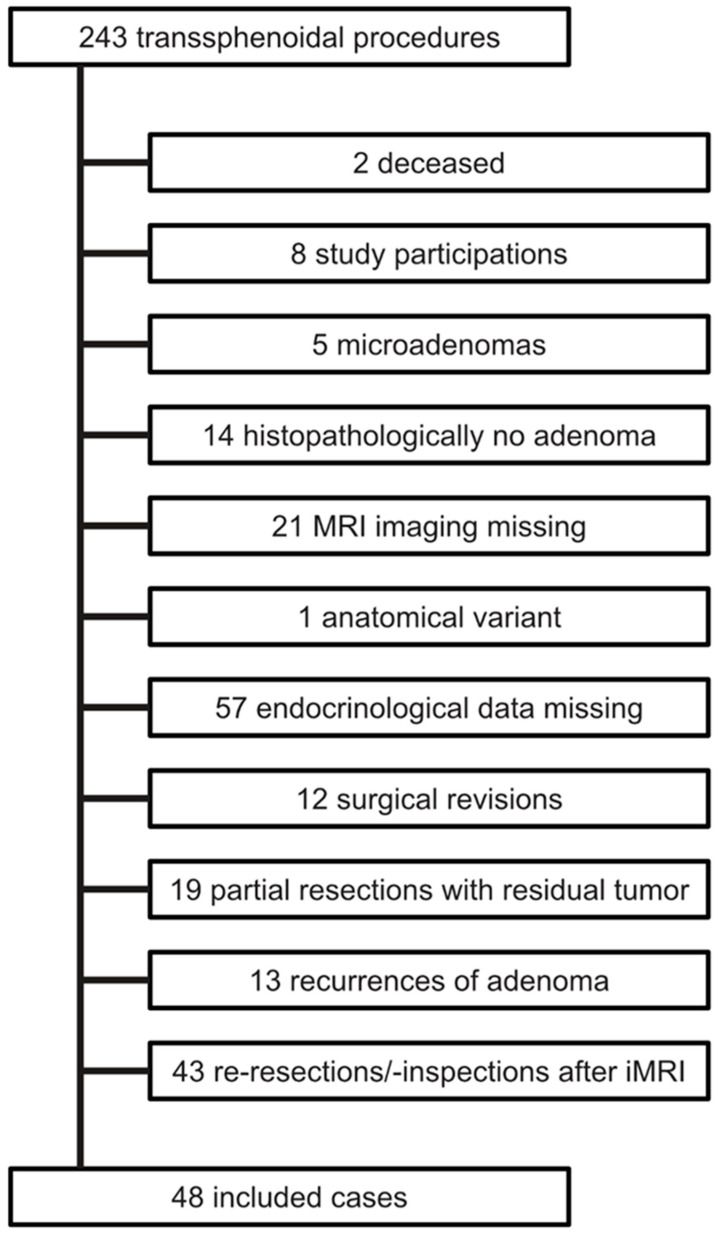
Flowchart illustrating the exclusion/inclusion process. The reasons for exclusion are listed in the order in which they were recorded; multiple reasons for exclusion per case are only mentioned once.

Forty-eight patients who underwent TSS for macroadenoma met the inclusion criteria and had sufficient radiological and endocrinological data available to be included in the final analysis. Detailed information on this process is shown in Figure 2. Because of a preoperatively prevalent adrenocortical insufficiency, the sample size was further reduced by 19 patients to a final sub-sample of 29 for the statistical analysis regarding new-onset adrenocortical insufficiency. None of these patients had Cushing disease or preoperative permanent AVP-D. In addition, no surgery was performed on prolactinomas. The mean age was 55 years, and there was an equal distribution of female and male patients. Knosp grades between 2 and 4 were most frequent. In addition, there was a strong correlation between the occurrence of AVP-D and NAI, with an exact contingency coefficient of 0.498 (*p* = 0.007). Further details are shown in Table 2.

### 3.2. New-Onset Adrenocortical Insufficiency

In total, 11 of 29 patients (38%) developed NAI in our study sample after excluding patients with preoperative adrenocortical insufficiency from the sub-analysis. The pituitary stalk diameter was 0.5 mm wider in patients with NAI on the same level as the insertion into the PG, the middle level, and the minimal value of the three acquired levels (*p* < 0.001 for diameter minimum). Detailed information can be found in Table 3. Following this, the binary logistic regression analysis was only performed for the minimal diameter and identified this parameter as a predictor of NAI with an odds ratio (OR) of 28.6 (*p* = 0.008). The Youden index identified 2.9 mm as the optimal cut-off for the minimal stalk diameter regarding NAI. Detailed statistical performance values can be found in Table 4.

A *t*-test for paired samples showed a statistically significant decline in pituitary stalk diameter on all levels of 0.6 to 0.7 mm between intraoperative and postoperative MRI after three months (*p* < 0.001 for diameter minimum). Further, in the follow-up MRI, there were no statistically significant differences in pituitary stalk diameters regarding NAI (*p* = 0.499 for diameter minimum). In addition, there was no correlation between pituitary stalk length and diameter (minimum) (Pearson correlation, *p* = 0.263) in iMRI.

### 3.3. (Transient) AVP-D

**Figure 3:**
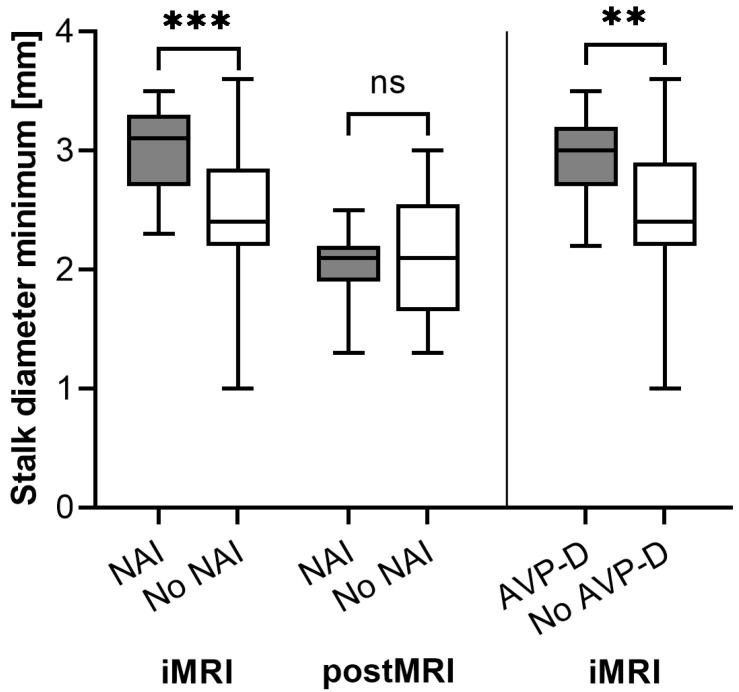
Differences in minimal sagittal pituitary stalk diameter in intraoperative (iMRI) and postoperative (post-MRI) MRI after 3 months regarding the prevalence of new-onset adrenocortical insufficiency (NAI) in *n* = 29 patients. The right side shows intraoperative (iMRI) differences in the minimal stalk diameter regarding arginine vasopressin deficiency (AVP-D) in *n* = 48 patients who underwent TSS (ns = not significant; ** = *p* < 0.01; *** = *p* < 0.001).

Twelve out of forty-eight patients (25%) showed transient AVP-D during the in-hospital stay, and one (2%) developed permanent AVP-D. Student’s *t*-test identified statistically significant differences in intraoperative sagittal pituitary stalk diameter on all three levels and the minimum value of these three (*p* = 0.008 for diameter minimum, Figure 3). On average, the stalk diameter was 0.4–0.5 mm greater in patients with new-onset AVP-D. Table 3 shows that no other group differences could be found. The binary logistic regression identified this variable as a predictor of AVP-D with an OR of 6.0 (*p* = 0.021). The Youden index identified 2.7 mm as the optimal cut-off for the minimal stalk diameter regarding AVP-D. Inter-imaging comparisons were not performed for AVP-D since AVP-D was only persistent in one patient.

## 4. Discussion

We conducted a retrospective monocentric study to identify (intraoperative) MRI pituitary stalk features predicting NAI and AVP-D.

### 4.1. Key Findings

In a retrospective analysis of 415 patients with pituitary adenomas undergoing TSS, Xue et al. found that more prominent PS deviation angles were associated with AVP-D after TSS (OR = 2.4, *p* = 0.004) [18]. However, we could not replicate these results for AVP-D or NAI (Table 3). Further, there was no difference in PS length regarding these two insufficiencies. According to the findings of Raveendranath et al., our results showing a mean pituitary stalk length (including the infundibular recess) of 10.8 (+/−2) mm are in good agreement with the findings in healthy subjects [19]. In addition, we could not identify other pituitary stalk aspects in MRI typical of brain tissue damage, such as T1 hypointensity and T2 hyperintensity, which are found in cerebral edema [20]. However, since these variables were obtained using iMRI, these typical findings might not have been present at the time of image acquisition.

A binary logistic regression model identified an OR of 6.0 for increased AVP-D frequency (*p* = 0.021). Therefore, 2.7 mm was determined to be a cut-off value of the minimal PSD for AVP-D. However, regarding AVP-D, several easy-to-acquire parameters, such as polyuria, negative fluid balance, decreased urine specific gravity, and osmolality with increased plasma sodium and serum osmolality, have already been established to determine this deficiency in postoperative inpatient settings [17,21]. Thus, clinically, the connection between iMRI findings and AVP-D seems even more relevant from a pathophysiological angle, as described below.

Unlike AVP-D, no optimal early postoperative diagnostics or therapies have been securely established for NAI [7,16]. Thus, there are ongoing efforts to use morning cortisol levels to predict NAI in the early postoperative course. Cerina et al. (2016) published the results of a prospective study enrolling 70 patients with PA. The onset of NAI was validated using the ITT 3–6 months postoperatively. They found that morning cortisol on the third postoperative day had the best predictive value for NAI with a sensitivity of 82.4% and a specificity of 83.3% in levels below 343 nmol/L (12.4 µg/dL). Further, morning cortisol on the third day was advantageous to morning cortisol on the sixth postoperative day. However, the predictive power could be significantly increased by adding tumor size, fT4, and TSH levels to the statistical model with a sensitivity of up to 94.1% and a specificity of 78.3% [22]. Polovina et al. performed early-morning cortisol testing on the second day after TSS by validating NAI via Synacthen^®^ testing three months post-surgery. Morning cortisol below an optimum cut-off of 300 nmol/L (10.9 µg/dL) was defined as NAI, with a sensitivity of 92% and a specificity of 87% [23]. In a comparative approach, Staby et al. found a basal cortisol cut-off concentration of 300 nmol/L (10.9 µg/dL) within 48 h of TSS to have a sensitivity of 100% and a specificity of 81% in their work-up on 143 cases who underwent TSS on PA. To validate NAI, a Synacthen^®^ test six months post-surgery was used [24]. Further, in a retrospective study, Butenschoen et al. analyzed 211 patients who underwent TSS for PA. Basal cortisol was measured on the fifth postoperative day. Butenschoen et al. found an optimal cut-off of 6.9 µg/dl with a sensitivity of 86% and a specificity of 56%. However, the same procedure six weeks after surgery and a cut-off of 6.95 µg/dL reached a sensitivity of 94% and a specificity of 68% for NAI, persisting one year after TSS [2]. In summary, the peer-reviewed literature shows that early postoperative morning cortisol achieves a comparatively high sensitivity between 82 and 100% and a specificity between 56 and 87% for predicting NAI after TSS for PA. However, differing optimal cut-off values of 6.9, 10.9, and 12.4 µg/dL on differing postoperative days are presented [2,22,23,24]. Furthermore, different therapy regimens have been used, with some centers establishing hydrocortisone substitution and some centers not beginning substitution unless basal cortisol levels show NAI.

Our analysis found that a thickening of the pituitary stalk (PST) in sagittal iMRI was associated with a higher frequency of NAI. We identified an optimal cut-off value of 2.9 mm for the minimal pituitary stalk diameter. Simultaneously, we found that this thickening was not accompanied by a shortening of the pituitary stalk or, thus, a compressed impression of the stalk (*p* = 0.263). Following this, intraoperative PST confirmed the frequent intraoperative impression of edematous thickening of the stalk. Since this parameter is easy to obtain in iMRI, high specificity and NPV seem to add an especially relevant benefit to the diagnostic security of NAI after TSS for pituitary macroadenomas, and they might help reduce unnecessary hydrocortisone substitution therapies.

### 4.2. Pathophysiological Considerations

However, a common pathomechanism seems conclusive because of the correlation between NAI and AVP-D in our study population and PST as predictive factors for both insufficiencies. There is an anatomical connection between AVP and CRH in the hypothalamic-pituitary axis in the blood supply [25,26]. In summary, the inferior hypophyseal artery (IHA) lends an arterial blood supply to the posterior PG and the PS. In contrast, the superior hypophyseal artery (SHA) supplies the median eminence; infundibulum; and, via portal vessels, the anterior PG [27,28]. In a radiologic study, Ito et al. found that the blood supply of larger adenomas (20–49 mm) was more frequently established by the internal carotid artery and IHA rather than the SHA [28]. This correlates well with our sample’s mean maximal adenoma diameter of 22 mm. Thus, anatomically, PST might be due to injuries to branches of the IHA, especially in enlarging adenoma with consecutive NAI and AVP-D.

Further, Staartjes et al. showed that early pruning (≥15% volume loss) in follow-up MRI after the early unfolding (≥15% volume gain) of the PG in iMRI was associated with new endocrinological deficits in 14% of patients and was a predictor of new-onset endocrinological insufficiency (OR 6.25, *p* = 0.024). However, PG volumes returned to preoperative levels in the MRI follow-up one-year post-surgery [29]. Our study found PST to be a predictive factor of AVP-D and NAI and a general decline of PSD of 0.6 to 0.7 mm in the control period three months post-surgery (*p* = 0.001). However, following the findings of Staartjes et al., PS thickening, like PG unfolding, might not always be detected in iMRI, and, thus, might only be detectable in early postoperative MRI [29].

### 4.3. Limitations

To the best of our knowledge, our study is the first to describe measurable intraoperative pituitary stalk thickening as a predictor of NAI and AVP-D. However, regarding the validity of these results, the retrospective, monocentric study design has to be considered. Still, strict requirements for the completeness of endocrinological and radiological data were followed to maintain the quality and significance of the results. In particular, following the work of Staartjes et al. describing the intraoperative unfolding of the pituitary gland, the exclusion of patients on whom resections of residual tumors were performed after iMRI seemed reasonable in order not to miss possible morphological changes of the pituitary stalk because of further manipulation [29]. At the same time, it must be accepted that this particular patient sample did not directly benefit from using iMRI. Regardless, the changes in the pituitary stalk were detectable only intraoperatively but not on follow-up MRI. A study including early postoperative MRI during the inpatient stay seems necessary to eliminate this limitation and make the results more applicable to widespread use in hospitals where there is no possibility of performing iMRI.

The transient and permanent AVP-D frequencies were within values encountered in the peer-reviewed literature [10]. However, the frequency of NAI in 11 out of 29 patients (38%) was higher than the rates reported in the literature of up to 18% [4,5]. Still, when determining the frequency of new-onset adrenocortical insufficiency in all patients with macroadenomas in our collective, the frequency of NAI was much lower at 15% (15 out of 94 with no preoperative adrenocortical insufficiencies). Further, the prospective TRANSSPHER study has shown increased NAI rates when using microscopic techniques, which were also included in our sample [4]. In addition, comparable to our study sample, Fatemi et al. found that new hypopituitarism occurs most frequently in patients with tumor sizes over 20 mm [30]. Still, since all patients included in this analysis underwent total adenoma resections when intraoperative MRI was performed, a more aggressive surgical approach might also play a role. Nevertheless, the accuracy of measuring the stalk diameter might be confounded by the mediocre spatial resolution of 1.5 T MRI TSE sequences, especially considering the pituitary stalk angle and consecutive geometric constraint in slice imaging in our study population [31,32]. However, significant differences in PSD values regarding AVP-D and NAI seem reliable, though there may be geometric inaccuracies. Further, since Ling et al. showed different thickening patterns in the PS in MRI, it seems rational to define the minimum value of the maximum diameters of the PS at three different levels to increase reliability [33].

## 5. Conclusions

The findings of this retrospective study indicate that the PST in iMRI might be a predictive factor of AVP-D and NAI after TSS for pituitary macroadenomas. Regarding its high specificity and negative predictive value, PST was particularly suitable for excluding NAI in our study population. This might help avoid the over-substitution of hydrocortisone in patients undergoing TSS. However, a prospective study validating these findings seems obligatory. Further, the results should be replicated with early postoperative MRI to detect phenomena, such as early pruning of the PS, and allow for a more widespread application of these findings in centers where it is not possible to perform iMRI.

## Figures and Tables

**Table 1:** Detailed overview of the intraoperative MRI variables of the pituitary stalk.

Variable	Plane/Sequence	Definition
Diameter chiasm (mm)	sagittal T1c ^1^	The maximal pituitary stalk diameter is perpendicular to the longitudinal axis of the stalk at the optic chiasm, the level of the insertion into the pituitary gland, and the middle level in between.
Diameter middle (mm)	sagittal T1c ^1^	
Diameter gland (mm)	sagittal T1c ^1^	
Diameter minimum (mm)	sagittal T1c ^1^	The minimal value of the three levels listed above.
Length (mm)	sagittal T1c ^1^	The length of the pituitary stalk from the inflection point to the chiasmatic recess and the insertion into the gland.
Coronal angle (°)	coronal T1c ^1^	The absolute value of the angle of the pituitary stalk against the vertical. The pivot point is the infundibulum.
T1 signal ratio	sagittal T1	The ratio of the average T1/T2 signal intensity of a seizable region of interest in the pituitary stalk divided by a seizable region of interest in the callosal body.
T2 signal ratio	sagittal T2	

^1^ T1 contrast-enhanced.

**Table 2:** Characteristics of the study sample.

Variable	Unit	Value
Females: Males	N (%)	23 (48): 25 (52)
Age	Years (+/− SD)	55 (+/−16)
Endoscopic: Microscopic	N (%)	36 (75): 12 (25)
Tumor size ^1^	mm (+/− SD)	22 (+/−6)
Knosp classification		
1	N (%)	3 (6)
2	N (%)	13 (27)
3	N (%)	21 (44)
4	N (%)	11 (23)
Concomitant prolactinemia	N (%)	20 (42)
Preoperative endocrinological status		
No insufficiency	N (%)	23 (48)
Isolated adrenocortical insufficiency	N (%)	10 (38)
Isolated growth hormone insufficiency	N (%)	5 (10)
Isolated gonadotropin insufficiency	N (%)	0 (0)
Mixed insufficiency	N (%)	10 (21) ^2^
New-onset adrenocortical insufficiency	N (%)	11 (38) ^3^
New-onset arginine vasopressin deficiency (AVP-D)		
Transient	N (%)	12 (25)
Permanent	N (%)	1 (2)

N = number; SD = standard deviation. ^1^ Largest diameter in any plane; ^2^ Nine patients with adrenocortical insufficiency; ^3^ Percentage is provided for a sub-sample of 29 patients with no preoperative adrenocortical insufficiency.

**Table 3:** Group differences in intraoperative MRI variables regarding new-onset adrenocortical insufficiency (NAI) and arginine vasopressin deficiency (AVP-D).

Variable	No NAI	NAI ^1^	*p*-Value	No AVP-D	AVP-D	*p*-Value
Diameter chiasm (mm)	3.5 (+/−0.6)	3.6 (+/−0.6)	0.253	3.3 (+/−0.7)	3.7 (+/−0.8)	0.032
Diameter middle (mm)	2.9 (+/−0.6)	3.4 (+/−0.7)	0.016	3.0 (+/−0.6)	3.4 (+/−0.7)	0.035
Diameter gland (mm)	2.7 (+/−0.7)	3.2 (+/−0.4)	0.028	2.7 (+/−0.6)	3.2 (+/−0.6)	0.007
Diameter minimum (mm)	2.5 (+/−0.4)	3.0 (+/−0.4)	<0.001	2.5 (+/−0.6)	2.9 (+/−0.3)	0.008
Length (mm)	10.5 (+/−1.4)	11.7 (+/−3.1)	0.245	10.7 (+/−1.8)	11.0 (+/−3.2)	0.789
Coronal angle (°)	13 (+/−14)	14 (+/−15)	0.869	13 (+/−12)	12 (+/−16)	0.416
T1 signal ratio	1.2 (+/−0.2)	1.2 (+/−0.3)	0.884	1.2 (+/−0.2)	1.1 (+/−0.1)	0.178
T2 signal ratio	2.6 (+/−0.6)	2.4 (+/−0.6)	0.339	2.5 (+/−0.5)	2.4 (+/−0.7)	0.461

All data are provided as mean +/− standard deviation; *p*-value ≤ 0.05. ^1^ Data are shown for *n* = 29 patients with no preoperative adrenocortical insufficiencies.

**Table 4:** Statistical performance measures for the determined cut-off values of the minimal pituitary stalk diameter regarding new-onset adrenocortical insufficiency (NAI) and arginine vasopressin deficiency (AVP-D).

Variable	Sensitivity	Specificity	PPV	NPV
Diameter minimum > 2.9 mm and NAI	73	89	80	84
Diameter minimum > 2.7 mm and AVP-D	92	66	50	96

NPV = negative predictive value; PPV = positive predictive value. All data are shown as percentages (%).

## Data Availability

The data presented in this study are available upon request from the corresponding author.
